# Transcriptomic analysis for the gamma-ray-induced sweetpotato mutants with altered stem growth pattern

**DOI:** 10.3389/fgene.2024.1419399

**Published:** 2024-07-31

**Authors:** Hyeong-Un Lee, Sangrea Shim, Mi Nam Chung, Taeyoung Lee, Won Park, Tae Hwa Kim, Kyo Hwui Lee, Koan Sik Woo, Sang-Sik Nam, Moon Young Kim, Suk-Ha Lee

**Affiliations:** ^1^ Department of Agriculture, Forestry and Bioresources and Research Institute of Agriculture and Life Sciences, Seoul National University, Seoul, Republic of Korea; ^2^ Bioenergy Crop Research Institute, National Institute of Crop Science, Rural Development Administration, Muan, Republic of Korea; ^3^ Department of Forest Resources, College of Forest and Environmental Sciences, Kangwon National University, Chuncheon, Republic of Korea; ^4^ Bioinformatics Institute, Macrogen Inc., Seoul, Republic of Korea; ^5^ Plant Genomics and Breeding Institute, Seoul National University, Seoul, Republic of Korea

**Keywords:** sweetpotato, gamma-ray-induced mutagenesis, stem development, RNA sequencing, small auxin up RNA (SAUR), phytochrome interacting factor 4 (PIF4), hormonal signaling

## Abstract

**Introduction:**

Sweetpotato faces breeding challenges due to physiological and genomic issues. Gamma radiation is a novel approach for inducing genetic variation in crops. We analyzed the transcriptomic changes in gamma ray-induced sweetpotato mutants with altered stem development compared with those in the wild-type 'Tongchaeru’ cultivar.

**Methods:**

RNA sequencing analyses were performed to identify changes in the expression of genes related to stem development.

**Results:**

Transcriptomic analysis identified 8,931 upregulated and 6,901 downregulated genes, including the upregulation of the auxin-responsive *SMALL AUXIN UP RNA* (*SAUR*) and three *PHYTOCHROME INTERACTING FACTOR 4* (*PIF4*) genes. *PIF4* is crucial for regulating the expression of early auxin-responsive *SAUR* genes and stem growth in *Arabidopsis thaliana*. In the mutant, several genes related to stem elongation, including *PIF4* and those involved in various signaling pathways such as auxin and gibberellin, were upregulated.

**Discussion:**

Our results suggest that gamma ray-induced mutations influence auxin-dependent stem development by modulating a complex regulatory network involving the expression of *PIF4* and *SAUR* genes, and other signaling pathways such as gibberellin and ethylene signaling genes. This study enhances our understanding of the regulatory mechanisms underlying stem growth in sweetpotato, providing valuable insights for genomics-assisted breeding efforts.

## 1 Introduction

Sweetpotato (*Ipomoea batatas*) is essential in global agriculture due to its nutritional value and adaptability to a wide range of climate conditions (Sapakhova et al., 2023; Pushpalatha and Gangadharan, 2024). However, the development of new sweetpotato varieties and the study of its genetics are impeded by infrequent flowering, self- and cross-incompatibilities, and a heterozygous hexaploid (2n = 6x = 90) structure with a large genome (2–3 Gb) (Jones, 1972; Varon et al., 1989; Ozias-Akins and Jarret, 1994; Gurmu et al., 2013). These traits complicate traditional breeding efforts, making it more challenging compared with the breeding of diploid crop species (Yan et al., 2022). Therefore, innovative breeding strategies that can overcome these barriers, unlock genetic diversity, and enhance the desirable traits of sweetpotato are needed.

Gamma radiation has been used to induce genetic variation in sweetpotato. Possessing potent mutagenic capabilities, gamma radiation can induce several types of genetic change, including direct and indirect DNA damage (Li et al., 2019; Hase et al., 2020; Du et al., 2022; Hirao et al., 2022). This method offers a promising approach for generating novel genetic variations, potentially leading to the development of sweetpotato varieties with improved growth patterns, abiotic stress tolerance, disease resistance, yield, and nutritional profiles (He et al., 2009; Shin et al., 2011; Zhang et al., 2017).

RNA sequencing of this complex genome sequence and performing gene annotation has helped identify genes linked to starch, anthocyanin, and carotenoid biosynthesis, and drought tolerance (Kou et al., 2020; Yoon et al., 2022; Liu et al., 2023). Recently, next-generation sequencing (NGS) technologies have been used to construct a haploid-resolved chromosome-scale *de novo* assembly of the autohexaploid sweetpotato genome (Yan et al., 2022; Yoon et al., 2022). Likewise, the reference genome sequence of sweetpotato could be used to help predicting the genetic factors associated with phenotypic alterations induced by mutagenesis.

In this study, we leveraged the sweetpotato reference genome to conduct RNA sequencing analysis. Our aim was to explore the complex and diverse genetic factors associated with the altered stem development in a novel sweetpotato mutant. Through RNA sequencing, we sought not only to identify the genetic factors governing the phenotypic changes induced by mutagenesis, but also to provide valuable information for genomics-assisted sweetpotato breeding.

## 2 Materials and methods

### 2.1 Plant materials and gamma ray treatment

Sweetpotato cultivar “Tongchaeru” was selected as the wild-type (WT) due to its significant agricultural utility and value in the Korean market (Lee et al., 2023). This cultivar exhibits excellent traits for vegetable use, such as high yield and nutritional components in leaves and petioles. Developed for vegetable use in 2020 by the Bioenergy Crop Research Institute, National Institute of Crop Science, its plant variety protection right was registered in 2021 at the SEED and VARIETY SERVICE with registration number 8788. It was also registered as germplasm (Accession ID: IT327308) at the National Agrobiodiversity Center, National Institute of Agricultural Sciences, Rural Development Administration.

To obtain the cuttings for gamma-ray treatment, storage roots of the WT were sown in the soil of a greenhouse at the Bioenergy Crop Research Institute, Muan, Republic of Korea in March in 2022. The stems of plants grown from storage roots in the greenhouse were cut to a length of approximately 30–40 cm. These cuttings underwent gamma-ray treatment in the low-level irradiation facility of the Advanced Radiation Technology Institute at the Korea Atomic Energy Research Institute, using a^60^Co source to administer a range of doses [0 (control), 25, 50, 75, 100, 150, 200, 250, and 300 Gy] for 7 h. The irradiated cuttings were cut into segments with two nodes each without leaves, and 150 node cuttings were planted in 50-cell plug trays across three replicates. The plants were cultivated for 60 days to determine the optimal irradiation dose based on the mortality rate.

Based on the suggested lethal dose 50 (LD50) for genetic mutation induction in plants (Ke et al., 2019; Kumar et al., 2020), we evaluated the reduction dose (RD) by exposing the plants to 0, 25, 50, 75, and 100 Gy of gamma radiation for 7 h. Plant height, leaf area, and total root length were measured for 20 plants per replicate to evaluate growth reduction, with each measurement repeated across three biological replicates. LD50 values were determined using sigmoid regression analysis, employing the curve_fit function from the SciPy library (v. 1.9.3). RD50 values were calculated using three-parameter logistic regression analysis with the LogisticRegression model from the scikit-learn library (version 1.1.3; www.scikit-learn.org). Both analyses were conducted using Python version 3.12.3 (www.python.org). Considering the LD50 and RD50, cuttings of WT were irradiated with 50, 100, 150 Gy gamma rays for 7 h, and the first mutant (M1V0) generation plants were excised into 1,426, 1,548, and 1,344 segments with two nodes each without leaves, respectively. These segments were planted in 50-cell plug trays. The M1V1 generation plants, generated from axillary buds of M1V0, were propagated to M1V3 through vegetative propagation, such as node cuttings, confirming the stable expression of the phenotypes. The M1V3 generation and WT plants were grown in individual pots under greenhouse conditions to observe phenotypic changes. The stem length and diameter of the mutant and WT plants were measured in M1V3 generation plants grown for 90 days after propagation (DAP).

### 2.2 RNA extraction, transcriptome sequencing, and RNA-seq analysis

Total RNA was extracted from the healthy stem tissue of three WT plants (WT-1, WT-2, and WT-3) and from three gamma ray-induced mutant plants (Mutant-1, Mutant-2, and Mutant-3) of M1V3 generation, grown for 30 DAP using Maxwell RSC Tissue, according to the manufacturer’s instructions. RNA integrity was evaluated using TapeStation RNA screentape (Agilent, #5067-5576), and only samples with an RNA integrity number (RIN) and 28S/18S rRNA ratio greater than or equal to 7 and 1, respectively, were used for RNA sequencing analysis.

Transcriptome sequencing (RNA-seq) was performed using the Novaseq6000 platform (Illumina, San Diego, United States). Libraries were prepared using Illumina TruSeq Stranded Total RNA Library Prep Plant Kit (Illumina, San Diego, CA, United States, #20020611), targeting a sequencing depth of 100 million reads per sample. The RNA-seq data were processed using Trimmomatic (v. 0.38; Bolger et al., 2014) to remove adapters and low-quality sequences, followed by mapping to the reference genome of sweetpotato using HISAT2 (v2.1.0; Kim et al., 2019). Based on the RNA-seq mapping result, gene expression value for high-confident gene model was calculated using StringTie (v. 2.1.3b; Pertea et al., 2015).

### 2.3 Differential gene expression analysis

Hierarchical clustering on the regularized log-transformed gene expression values for the whole reference gene set was performed with the following parameters: distance metric = Euclidean distance, linkage method = complete. Principal component analysis (PCA) and heatmap analysis were conducted to confirm the similarity between samples. Differential gene expression analysis was performed using the DESeq2 R package (Love et al., 2014), with significant changes defined by statistical significance (*p* < 0.01) and fold change (|log2FC| ≧ 1) thresholds. We compared the results of using this criterion (*p* < 0.01) to those obtained using adjusted *p*-values with the Benjamini-Hochberg procedure (adjusted *p* < 0.05) for differential gene expression. The expression pattern of DEGs per sample was visualized using a heatmap. To explore the underlying biological meaning of the genes differentially expressed, we employed functional enrichment analysis based on Gene Ontology (GO, Consortium, 2019) and Kyoto Encyclopedia of Genes and Genomes annotations (KEGG, Kanehisa et al., 2016). The GO terms were annotated using InterproScan with “-goterms” parameter (v. 5.34-73.0; Jones et al., 2014). The KEGG pathway of the whole sweetpotato reference transcriptome was annotated using the Assign KO tool in the KEGG mapper (https://www.kegg.jp/kegg/mapper/assign_ko.html) and the Map pathway tool in the KEGG Orthology database (https://www.kegg.jp/kegg/ko.html). Statistical significance of enrichment analysis was determined using the Python hypergeometric test module [scipy.stats.hypergeom.sf] (Virtanen et al., 2020). In detail, the number of the entire reference gene set, the reference genes for a specific annotation category, the genes of interest, and the genes of interest for a specific annotation category were counted and used for the hypergeometric test. All data analysis and visualization of the differentially expressed genes were conducted using R 3.6.1 (www.r-project.org), DESeq2 R package (Love et al., 2014), and Python Seaborn module (Waskom, 2021).

## 3 Results

### 3.1 Induction and selection of gamma ray-induced sweetpotato mutants

**FIGURE 1:**
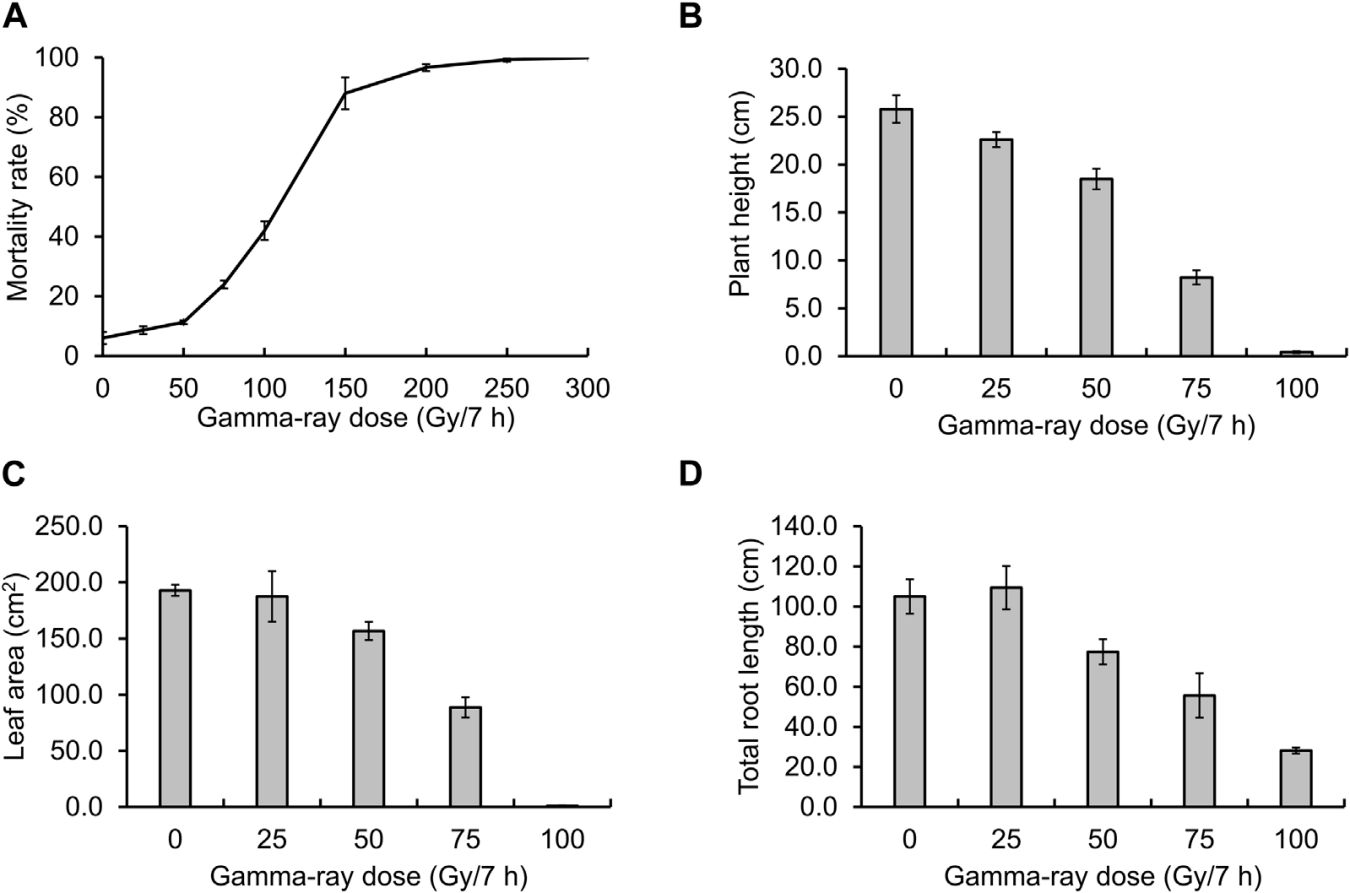
Optimal gamma-ray dosage determination. The optimal dosage of gamma radiation was determined by examining the mortality rate **(A)** and the impact on plant growth, including the reduction in height **(B)**, leaf area **(C)**, and total root length **(D)** across different gamma-ray dosages.

**FIGURE 2:**
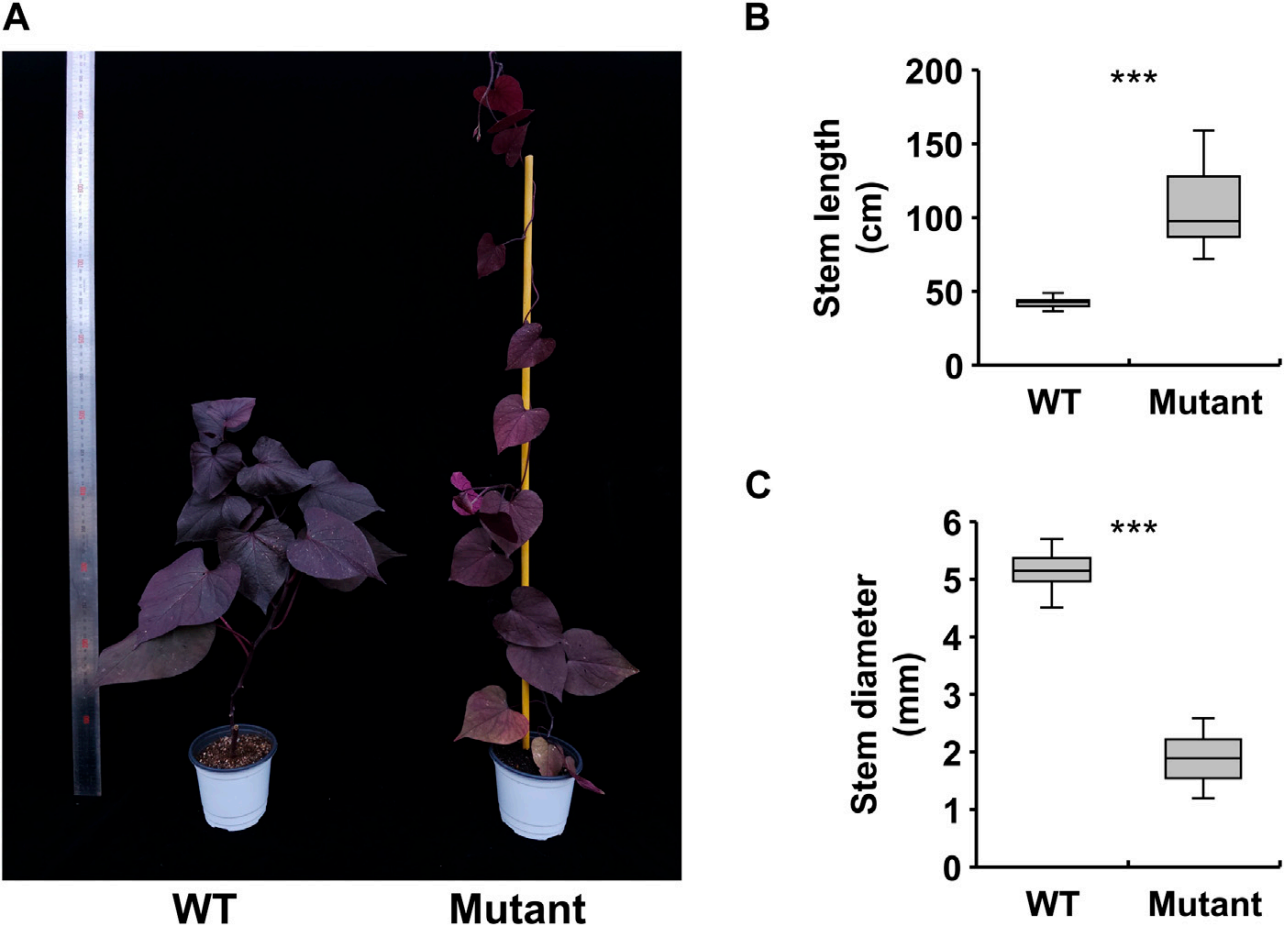
Phenotypic variations in a gamma-ray-induced sweetpotato mutant. Comparison of the phenotype of a gamma-ray-induced mutant to that of the wild-type (WT) (**A**) and measurement of stem growth patterns, including length and diameter (**B, C**). Statistical significance was determined by a one-tailed *t*-test with unequal variance and is indicated by asterisk marks (*p* < 0.001).

Before creating gamma ray-induced sweetpotato mutants, we established the optimal dosage of gamma rays by monitoring the rate of lethality and growth reduction. The lethality rate increased in a sigmoid pattern and the estimated LD50 value was approximately 105.4 Gy/7 h (Figure 1A). The RD50 values estimated from plant height, leaf area, and total root length were 63.9 Gy, 71.8 Gy, and 72.7 Gy, respectively (Figures 1B–D). The optimal gamma ray dosage range to induce mutations in sweetpotato cultivar “Tongchaeru” was 63.9–105.4 Gy over 7 h. Based on this optimal dosage of gamma rays, we treated WT stem cuttings with doses of 50, 100, and 150 Gy for 7 h. Among the 1,548 M1V0 generation plants treated with 100 Gy of gamma radiation, a mutant with stems distinctly thinner and longer than those of the WT was selected for further study (Figure 2A). This mutant exhibited a significant and stable phenotypic variation in stem length and diameter in M1V3 compared with WT plants (Figures 2B, C).

### 3.2 Global transcriptional changes caused by gamma-ray induced mutation

To understand the transcriptional regulation of gamma ray-induced mutations, we conducted RNA-seq on the stem tissues of WT and mutant plants. We generated reads for each library and trimmed them for sequence alignment. On average, 93% of the trimmed reads were successfully mapped to genes in the sweetpotato genome (Table 1).

**TABLE 1:** Summary statistics for RNA sequencing and mapping.

Genotype	Biological replication	No. of total reads	No. of trimmed reads	No. of mapped reads (%)
Wild-type	1	98,670,626	96,680,006	90,934,680 (94.06)
	2	100,277,496	98,431,562	92,060,766 (93.53)
	3	104,898,308	103,092,508	97,425,703 (94.50)
Mutant	1	106,161,182	104,328,468	96,584,818 (92.58)
	2	107,673,568	105,841,656	97,857,994 (92.46)
	3	129,224,668	126,976,280	117,886,627 (92.84)

**FIGURE 3:**
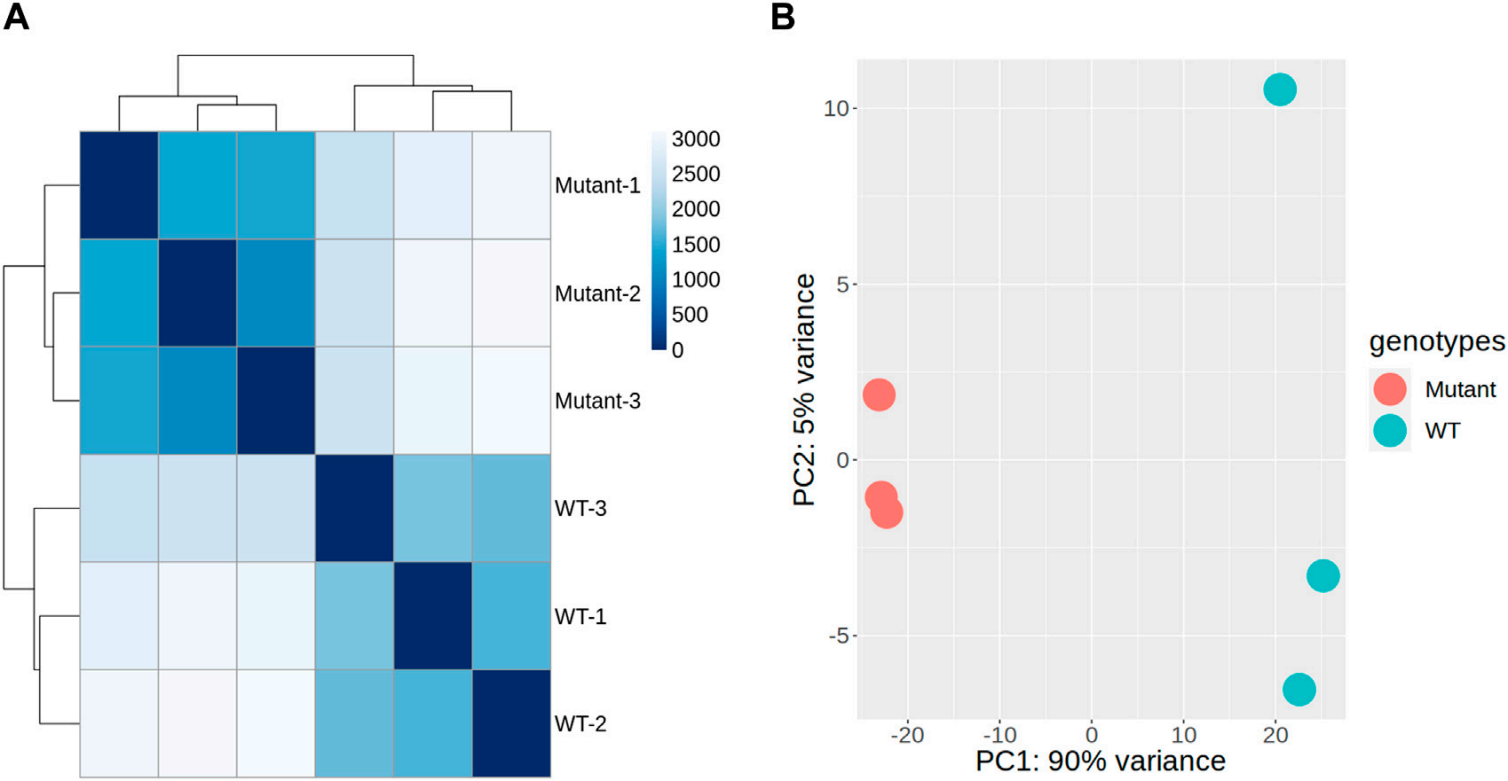
Genome-wide transcription patterns in gamma-ray-induced mutant and wild-type (WT) sweetpotato. Correlogram of samples with hierarchical clustering **(A)** and principal component analysis (PCA) plots **(B)**, illustrating the transcriptional variance among the samples, with PC1 accounting for 90% of the variance and reflecting the primary differences in gene expression profiles between mutant and WT plants. PC2 explains an additional 5% of the variance and may capture variations that are not explained by PC1.

To assess the impact of gamma radiation-induced mutations on overall gene expression, we conducted hierarchical clustering and PCA on RNA-seq data derived from the stem tissues of mutant and WT plants. Hierarchical clustering demonstrated a consistent expression pattern among the WT samples, indicating uniformity across the control samples (Figure 3A). In contrast, the mutant samples displayed distinct expression profiles, forming separate clusters. This transcriptional divergence suggests that the mutations induced by gamma radiation significantly affect gene expression in stem tissues (Figure 3A). The distances between clusters in the dendrogram underscored the homogeneity within each group and the pronounced differences between WT and mutant samples, indicating the substantial influence of mutations on gene expression patterns and alterations in the regulatory mechanisms within the mutants.

PCA further confirmed these findings, with the first principal component (PC1) along the x-axis explaining 90% of the total variance, indicating a major shift in gene expression predominantly associated with genotypic differences (Figure 3B). The second principal component (PC2) along the y-axis captured an additional 5% of the variance, further distinguishing the variation within each genotype. This analysis implied that the mutations introduced a higher degree of expression variability within the mutant. Consequently, we performed a comparative gene expression analysis between the mutant and WT samples to identify specific transcriptional changes.

### 3.3 Analysis of differentially expressed genes in gamma ray-induced mutant sweetpotato

**FIGURE 4:**
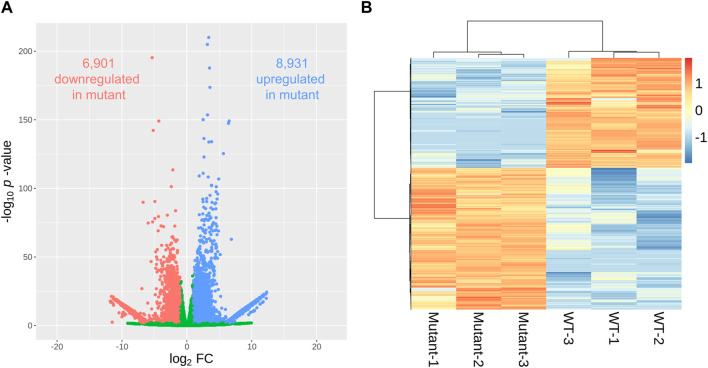
Differentially expressed genes (DEGs) in gamma-ray-induced mutants and wild-type (WT) sweetpotato. The pattern of DEGs is visualized in a volcano plot **(A)**, where the x- and y-axes represent the log_2_ fold change and statistical significance (*p*-value), respectively. Red and blue dots indicate significantly down- and upregulated DEGs (|log_2_FC| ≧1 and *p*-value < 0.01), respectively. The expression pattern of DEGs per sample is visualized in a heatmap **(B)** using the log_10_ (normalized expression value + 1) for heatmap generation.

A comparative examination of gene expression between WT and mutant sweetpotato samples revealed a wide array of transcriptional changes. We found that the genes selected with the *p*-value <0.01 criteria were less than adjusted *p*-values <0.05. Using a volcano plot to illustrate the relationship between the fold change and statistical significance for each gene, we identified 15,832 genes exhibiting significant differential expression (*p* < 0.01) (Figure 4A). Among these, 8,931 and 6,901 genes in the mutant were up- and downregulated, respectively, compared to those in the WT (Supplementary Tables S1, S2). The hierarchical clustering of differentially expressed genes (DEGs) presented in the heatmap further delineates the distinct gene expression profiles between the WT and mutant samples (Figure 4B), reinforcing the substantial transcriptional divergence caused by gamma-ray-induced mutations. The clustering pattern showed tight grouping within each genotype, highlighting consistent expression within biological replicates and marked differences between genotypes.

### 3.4 Pathway and functional enrichment analyses of mutation-affected genes and differentially expressed genes

**FIGURE 5:**
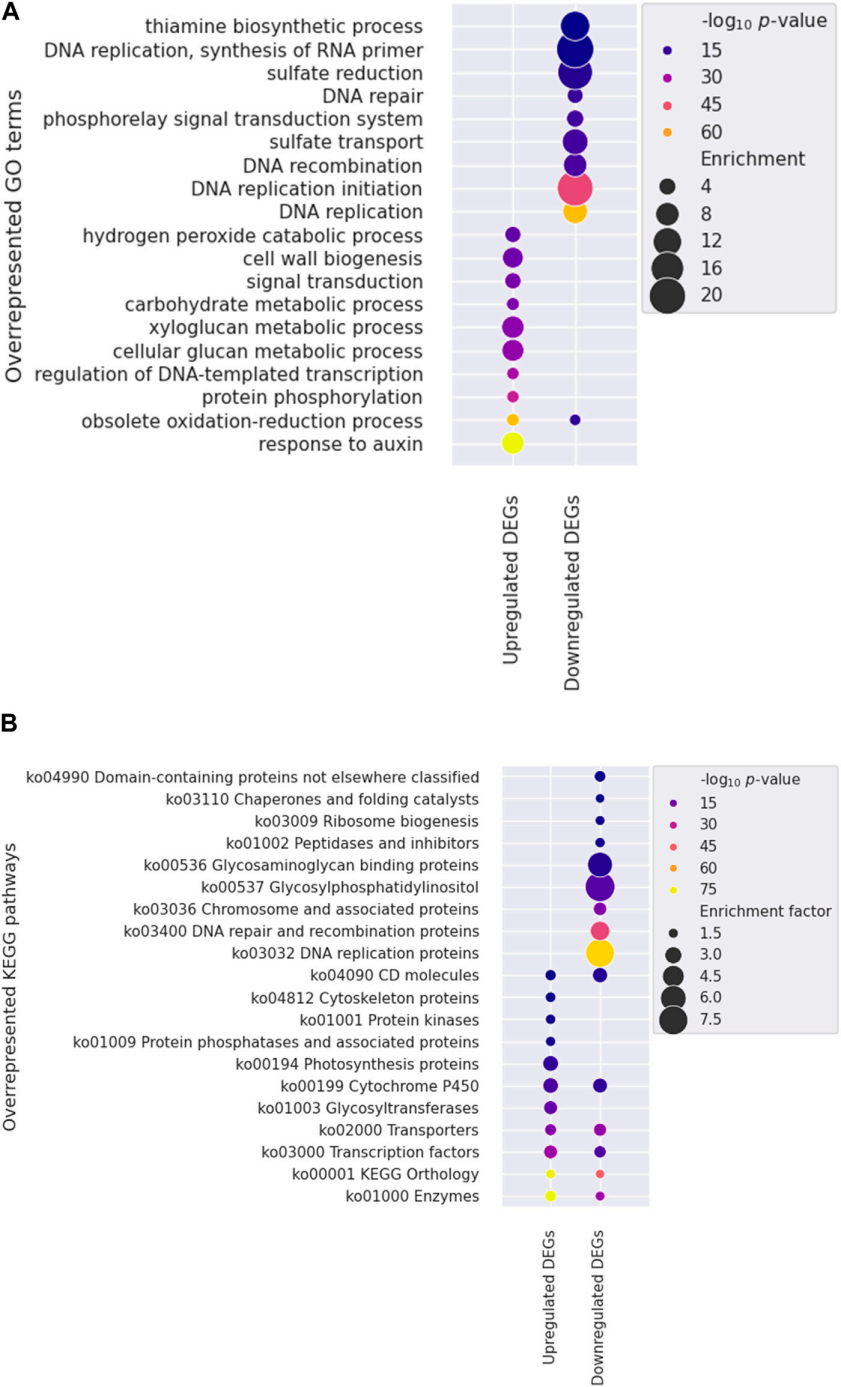
Functional enrichment analysis utilizing Gene Ontology (GO) and Kyoto Encyclopedia of Genes and Genomes (KEGG) annotations. Overrepresented GO terms **(A)** and KEGG pathways **(B)** are shown. The color and size of each dot indicate the statistical significance (log_10_ *p*-value) and enrichment factor, respectively. Note that the ten most significantly overrepresented terms in the biological process category were presented for each gene set in the GO analysis.

To identify specific factors influencing stem growth among the various factors in the mutants, we conducted gene ontology (GO) and KEGG pathway enrichment analyses to show changed gene expression. One significant finding was the over-representation of genes associated with the response to auxin (GO:0009733) among the upregulated DEGs (Figure 5A; Supplementary Table S3), with 114 of 129 genes being *SMALL AUXIN UP RNA* (*SAUR*) genes. Auxin, gibberellic acid (GA), and brassinosteroid (BR) are primary hormones that significantly enhance plant growth (Choi and Oh, 2016). Auxin-related AUXIN RESPONSE FACTOR (ARF6), BR-related BRASSINAZOLE RESISTANT 1 (BZR1), and PHYTOCHROME INTERACTING FACTOR 4 (PIF4) share many target genes and work together to regulate the expression of genes involved in cell elongation, such as *SAURs* (Oh et al., 2014).

Our results showed significant upregulation of several genes associated with auxin signaling and response. Notably, genes related to auxin signaling such as *AUX/IAA domain*, *ARF*, *YUCCA* (*YUC*) were upregulated in our mutant. Auxin regulates transcription by binding to AUXIN/INDOLE-3-ACETIC ACID (Aux/IAA) proteins and F-box proteins of the TRANSPORT INHIBITOR RESPONSE 1/AUXIN SIGNALING F-BOX (TIR1/AFB) family, leading to the degradation of Aux/IAAs and the subsequent release of repression at AUXIN RESPONSE ELEMENT (ARE)-containing promoters (Leyser, 2018). This process involves the polyubiquitination and degradation of Aux/IAAs, allowing ARF protein dimers to activate gene expression. In our mutant, the expression of *ARF4-like isoform X1* and *ARF19-like* was increased, whereas *ARF6* interacting with PIF4 was not upregulated. Additionally, auxin biosynthesis-related genes, including *YUC5 and YUC10*, were upregulated in our mutant. *YUC* genes play a crucial role in auxin biosynthesis by catalyzing a key step in the conversion of tryptophan to indole-3-acetic acid (IAA), the primary form of auxin (Cao et al., 2019; Wu et al., 2023). *YUC5* is expressed during root development, while *YUC10* is associated with embryogenesis (Cheng et al., 2007; Song et al., 2020). PIF4 directly upregulates the expression of *YUC8*, thereby increasing auxin biosynthesis and promoting hypocotyl elongation in response to high temperatures (Sun et al., 2012).

**FIGURE 6:**
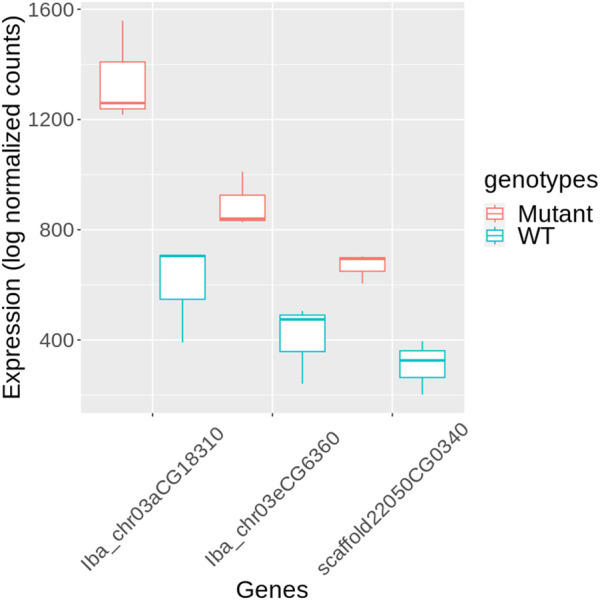
Expression of PIF4 genes in mutant and wild-type (WT) sweetpotato. Expression levels are presented as log normalized counts. Red box plots represent the mutant samples, and blue box plots represent the WT samples. The genes shown are *Iba_chr03aCG18310*, *Iba_chr03eCG6360*, and *scaffold22050CG0340*.

KEGG annotation revealed that genes within categories such as enzymes (ko01000), TFs (ko03000), and transporters (ko02000), were predominantly overrepresented among DEGs (Figure 5B, Supplementary Table S4). We further investigated the TFs to identify potential candidates regulating stem development in mutant plants. Three genes encoding PIF4 were notably upregulated in mutant plants (Figure 6), and these TF genes are crucial for stem elongation in *Arabidopsis thaliana* (Choi and Oh, 2016; Pereyra et al., 2023). However, the expression of *BZR1*, another important TF gene involved in stem elongation, was not upregulated in our mutant.

Gibberellin (GA) facilitates the elongation of the hypocotyl by causing the degradation of DELLA proteins, which are negative regulators (Choi and Oh, 2016). DELLA proteins bind directly to the basic helix-loop-helix (bHLH) DNA binding motif of PIF4, inhibiting its ability to attach to target gene promoters and thus rendering PIFs inactive (De Lucas et al., 2008; Feng et al., 2008; Leivar and Monte, 2014). In the GA biosynthesis and signal transduction pathway, *GIBBERELLIN 20-OXIDASE* (GA20ox) enzymes are essential for producing bioactive GAs. These enzymes transform GA precursors into active GAs, which are crucial for processes such as stem elongation, seed germination, and various other growth activities (Yamaguchi, 2008; Hedden and Thomas, 2012). *GIBBERELLIN INSENSITIVE DWARF1* (GID1) functions as a GA receptor that, upon binding with GA, facilitates the degradation of DELLA proteins through SKP1-CULLIN-F-BOX PROTEIN (SCF) ubiquitin E3 ligase-mediated ubiquitination (Colebrook et al., 2014). In our mutant, *gibberellin 20 oxidase 1-D-like* and *gibberellin receptor GID1B-like* were upregulated (Supplementary Table S5), which is considered to contribute to PIF4 activation due to DELLA destabilization.

Regarding ethylene, the expression of *ethylene-responsive transcription factor* was upregulated in our mutant (Supplementary Table S5). Ethylene-responsive transcription factors have been recognized as key regulators in the elongation of internodes induced by submergence (Hattori et al., 2009). Expansins are proteins that play a role in loosening plant cell walls, facilitating cell enlargement and a range of developmental processes that involve cell wall modification (Cosgrove, 2000). In our mutant, *Expansion-A10-like, Expansion-A2-like*, and *Expansion-A4-like* were upregulated (Supplementary Table S5). *SMAX1-Like 6-like* and *SMAX1-Like 7-like* are known to promote the branching of shoots while simultaneously inhibiting the elongation of petioles (Zheng et al., 2021). In our mutant, the expression of *SMAX1-Like 3*, *SMAX1-Like 6-like*, *SMAX1-Like 7-like* was downregulated (Supplementary Table S6).

## 4 Discussion

The genetic improvement of sweetpotato through traditional breeding methods is impeded by intrinsic biological characteristics, including infrequent flowering, self- and cross-incompatibility barriers, and a complex heterozygous hexaploid genome (2n = 6x = 90) that spans approximately 2–3 Gb (Jones, 1972; Varon et al., 1989; Ozias-Akins and Jarret, 1994; Gurmu et al., 2013). To circumvent these obstacles, mutagenesis using gamma irradiation has been widely used in sweetpotato breeding (He et al., 2009; Shin et al., 2011; Zhang et al., 2017). With the availability of the sweetpotato reference genome (Yoon et al., 2022), we aimed to explore genome-wide transcriptional alterations resulting from gamma ray-induced mutations using RNA-seq. By conducting transcriptome analyses of the WT and mutant lines, we were able to identify the putative genetic factors governing the altered stem growth patterns in sweetpotato mutants.

In our GO analyses, we identified that the “response to auxin” is a significant pathway influencing stem growth in the mutants. Furthermore, the upregulation of *SAUR* genes, which are directly regulated by *PIF4* and function in auxin-mediated growth responses in *A. thaliana* (Ren and Gray, 2015; Pereyra et al., 2023), provides a clearer understanding of the auxin-dependent molecular mechanisms that influence stem development in sweetpotato. Moreover, our mutants also showed increased expression of GA biosynthesis-related *GA20ox* and GA receptor gene *GID1*, which inhibit DELLA proteins that inactivate PIF4. Additionally, the upregulation of ethylene-responsive transcription factors and expansin genes, along with the downregulation of SMAX1-like genes, highlights the complex regulatory network of various hormonal and genetic factors influencing stem growth.

PIF4 promotes cell elongation and its activity is influenced by a range of environmental signals such as light and temperature, as well as hormonal signals including auxin, gibberellic acid, and brassinosteroid, through both transcriptional and post-translational mechanisms (Choi and Oh, 2016). PIF4 facilitates the removal of the repressive histone variant H2A.Z at thermo-responsive genes by interacting with the INO80 chromatin remodeling complex, which enhances gene expression (Kumar and Wigge, 2010; Willige et al., 2021). Additionally, PIF4 forms complexes with histone acetyltransferases (HAM1/2) and methylation readers (MRG1/2), promoting histone acetylation and subsequent gene expression in response to environmental cues such as shade and heat (Peng et al., 2018; Zhou et al., 2024). These interactions highlight the intricate network through which PIF4 influences plant development in response to variations in light and temperature (Ammari et al., 2024). The involvement of histone modifications and chromatin remodeling underscores the epigenetic regulation of PIF4 activity.

Recently, *IbPIF4* in sweetpotato has been reported to be significantly upregulated under abiotic stresses such as salt, H_2_O_2_, and heat, as well as biotic stresses including Fusarium wilt and stem nematode infections (Nie et al., 2023). Our transcriptomic approach utilizing a gamma-ray-induced mutant with upregulated PIF4 gene expression is expected to make a significant contribution to identifying genetic factors related to various stress responses and improving sweetpotato cultivars in further studies. Such investigative efforts promise to deepen our understanding of the role of *PIF4*, thereby enabling the development of innovative strategies for the genetic enhancement and breeding of sweetpotato varieties with desirable traits.

In future research, we need to investigate the relationship between polymorphism and the transcriptional regulation of genes related to stem elongation, particularly in polyploid crops such as sweetpotato. Polyploids possess multiple homoeo-alleles distributed across homoeologous chromosomes, leading to complex inheritance patterns that complicate the accurate estimation of allele dosages (Dufresne et al., 2014). Accurate SNP detection in polyploid crops is challenging due to the ambiguous alignment of highly similar homoeologous loci, which increases the rate of false positives in SNP identification (Clevenger and Ozias-Akins, 2015). The possible mechanism linking DNA variation and gene expression could involve changes in the DNA sequence at transcription factor binding sites and/or histone modifiers and could include other regulatory elements. These studies can provide insights into the possible mechanisms regulating gene expression in polyploid crops. Future research should delineate the specific molecular pathways through which key regulatory genes modulate stem growth in sweetpotato. This may involve conducting comprehensive functional analyses, including gene silencing or overexpression experiments, to evaluate the effects of these genes on organ development. Exploring the interactions between PIF4, other key regulatory genes, signaling pathways, and growth regulators is crucial for unraveling the multifaceted regulatory networks that influence organ development in sweetpotato.

Our study emphasizes the interactions between *PIF4* and *SAUR* genes' expression, along with other significant factors related to hormonal signaling, in influencing the morphology of a sweetpotato mutant with altered stem growth patterns. Our results are expected to broaden the understanding of the regulatory mechanisms underlying stem growth, which is a significant agricultural trait in sweetpotato.

## Data Availability

The data presented in the study are deposited in the NCBI repository, accession number PRJNA1137316.
